# The heart-brain axis: unraveling the interconnections between cardiovascular and Alzheimer’s diseases

**DOI:** 10.3389/fcvm.2025.1685461

**Published:** 2025-11-24

**Authors:** Aili Toyli, Anjum Shaik, Chen Zhao, Qing-Hui Chen, Qiuying Sha, Weihua Zhou

**Affiliations:** 1 Department of Mathematical Sciences, Michigan Technological University, Houghton, MI, United States; 2 Department of Applied Computing, Michigan Technological University, Houghton, MI, United States; 3 Department of Computer Science, Kennesaw State University, Marietta, GA, United States; 4 Department of Kinesiology and Integrative Physiology, Michigan Technological University, Houghton, MI, United States; 5 Center for Biocomputing and Digital Health, Institute of Computing and Cyber-systems, and Health Research Institute, Michigan Technological University, Houghton, MI, United States

**Keywords:** heart-brain axis, cardiovascular disease, Alzheimer's disease, imaging, dementia

## Abstract

Cardiovascular disease (CVD) and Alzheimer's disease (AD) are leading causes of death and disability worldwide, and recent research has increasingly illuminated a complex, bidirectional relationship between the two. This review synthesizes epidemiological, mechanistic, imaging, and genetic evidence linking CVD and AD through the heart-brain axis—a network of interrelated physiological and demographic pathways. We detail how cerebral hypoperfusion, inflammation, blood-brain barrier dysfunction, imbalance of the autonomic nervous system, and systemic amyloidosis contribute to shared neurodegenerative and cardiovascular outcomes. Multi-organ imaging studies, including MRI and PET, reveal that dysfunction of the cardiovascular system correlates with brain atrophy, white matter lesions, glymphatic impairment, and accumulation of AD-related proteinopathies. Genetic analyses further support overlapping risk architectures, particularly involving APOE and loci associated with lipid metabolism, vascular integrity, and inflammation. Age and sex are critical modifiers, with midlife CVD exerting the strongest influence on later cognitive decline, and sex-specific physiological responses shaping disease susceptibility. Finally, we explore how modifiable lifestyle factors, pharmacologic interventions, and precision medicine approaches targeting inflammatory and vascular pathways can jointly reduce the burden of both CVD and AD. Multidisciplinary collaboration to understand the interconnected biology of the heart and brain is essential for advancing integrated prevention and treatment strategies in aging populations.

## Introduction

Cardiovascular disease (CVD) and Alzheimer's disease (AD) are among the most pressing health challenges globally, significantly contributing to mortality and morbidity. CVD encompasses conditions such as coronary heart disease (CHD), stroke, and heart failure, which collectively are among the leading causes of death worldwide, claiming approximately 17.9 million lives annually (1). In the United States, CVD remains the foremost cause of death, with millions at risk due to hypertension, atherosclerosis, and obesity.

AD is a progressive neurodegenerative disease resulting in cognitive decline and neuropathologically characterized by the development of β-amyloid (Aβ) plaques and neurofibrillary tau tangles (2). It is the predominant form of dementia, affecting 6.9 million individuals in the United States and over 50 million globally (3). The number of AD cases is expected to nearly double by 2060 due to aging populations (4). Beyond the personal toll on patients and families, the societal burden is immense, with unpaid care for dementia valued at $346.6 billion in 2023 (3).

Initially regarded as distinct conditions, emerging research has illuminated critical interconnections between CVD and AD. Mechanisms such as atherosclerosis, hypertension, and inflammation are implicated in AD progression, likely through pathways involving vascular damage that impairs cerebral blood flow and promotes neurodegeneration. Conversely, AD-associated neurodegeneration can disrupt autonomic nervous system regulation, exacerbating cardiovascular issues. This bidirectional communication underscores the potential preventative value of managing CVD risk factors to mitigate AD risk and vice versa.

Shared risk factors further complicate this relationship. Age is a significant risk factor for both diseases, with CVD prevalence highest among those aged 65 and older and dementia incidence rising with age (4). Lifestyle factors including smoking, high-fat diets, physical inactivity, and excessive alcohol consumption elevate the risk for both CVD and AD (5). Additionally, intermediate metabolic factors such as hypertension, diabetes, and high blood lipid levels are well-documented contributors to CVD and are associated with increased AD risk. Biomarkers like inflammatory cytokines and neuroimaging findings reinforce the shared biological pathways predisposing individuals to both conditions (6).

Advanced imaging technologies have significantly enhanced our understanding of the heart-brain axis. Techniques such as magnetic resonance imaging (MRI) and positron emission tomography (PET) provide insights into the structural and functional interplays between the cardiovascular and central nervous systems (7).

This narrative review aims to comprehensively present hypothesized functional pathways linking CVD and AD. While other reviews have provided insight into the heart-brain axis, we expand up on their research by providing a thorough review of the imaging and genetic evidence for mechanistic pathways linking the heart and the brain. We will begin by highlighting clinical associations observed between AD and CVD. Next, we will elucidate the biological pathways forming the heart-brain axis. This will be followed by a discussion of evidence for the heart-brain axis derived from imaging studies. The next section will delve into the genetic framework supporting the heart-brain axis, focusing on variants contributing to both CVD and AD. We subsequently will investigate demographic interactions within the heart-brain axis. Finally, the clinical implications of this relationship will be examined, with particular emphasis on the potential for targeted therapeutic strategies aimed at preventing both cardiovascular and neurodegenerative diseases. By providing this overview of heart-brain pathways and the evidence supporting them, this paper aims to shed light on the complex interplay between AD and CVD and the implications for prevention and treatment of these diseases.

## Epidemiological overlap

A growing body of longitudinal evidence indicates a robust link between CVD and AD (8). In the Cardiovascular Health Study, Newman et al. reported AD incidence rates of 34.4 per 1000 person-years in individuals with prior CVD, vs. only 22.2 in those without (9). Similarly, the Whitehall II cohort showed that a higher Life's Simple 7 cardiovascular health score at age 50 predicted a lower subsequent dementia risk (10). Machine-learning approaches yielded comparable findings identifying accelerated CVD risk trajectories in late life as strong predictors of AD relative to stable trajectories  (11). Together, these studies support a robust association between CVD and AD.

This overlap is partially driven by shared modifiable risk factors such as hypertension, dyslipidemia, smoking, lack of exercise, and depression. Up to one-third of AD-related dementias may be attributable to cardiovascular risk profiles (12). The 2020 *Lancet* commission listed twelve intervention targets for dementia prevention, many of which, including diabetes, midlife obesity, alcohol misuse, and smoking, are also classic cardiac risk factors (13). This overlap complicates causal inference, raising questions regarding the role of CVD as an upstream driver of AD, a parallel manifestation of common exposures, or both.

Smoking is a major modifiable risk factor for both CVD and AD, with compelling evidence linking it to an increased risk of both conditions. In a large prospective cohort, smokers had more than double the risk of AD compared with never-smokers [Relative Risk (RR) 2.3]  (14). Research from the Northern Finland Birth Cohort 1966 linked smoking to adverse lipid profiles and higher antihypertensive and statin use  (15). Such evidence provides an example underscoring how single behaviors amplify pathology in both organs.

Several subtypes of CVD have been independently associated with increased risk for AD and other forms of dementia, one of which is heart failure (HF). In a population-based cohort study of individuals aged 75 years or older, HF was associated with an increased risk of all-cause and AD-specific dementia [Hazard Ratio (HR) 1.84 and HR 1.80, respectively] (16). This association was found to be robust across multiple populations, as a meta-analysis found HF to elevate the risk of AD by 53% across six studies (17). Hypertension's role as a shared risk factor may explain the relationship between HF and AD, as the use of antihypertensive drugs, primarily diuretics, was found to reduce the risk of dementia due to HF (HR 1.38) (16).

CHD occurs when plaque buildup in the arteries results in insufficient delivery of oxygen-rich blood to the heart muscle (18). Sun et al. found CHD to be associated with increased risk of AD with a random effects model [Odds Ratio (OR)/Risk Ratio (RR) 1.22] (17). A more recent prospective cohort study from the UK Biobank confirmed these findings, detecting significantly higher risks of developing all-cause dementia (HR 1.36), AD (HR 1.13), and vascular dementia (HR 1.78) in participants with CHD.

Another type of CVD firmly connecting the heart and brain is stroke. Stroke has been found to double the overall risk of dementia (19). Stroke has a unique role in the heart-brain axis, as it results in direct cerebral damage. Historically, dementia following incidence of stroke has been classified as vascular dementia, but growing evidence supports mixed vascular dementia and AD pathologies (20, 21). This overlap makes determining the differing effects of these pathologies difficult following stroke. Not only does stroke appear to increase the risk of AD, but conversely, the risk of stroke may be elevated in AD patients. A meta-analysis from Pinho et al. found the incidence rate for stroke to be significantly higher in AD patients than matched controls, primarily driven by intracerebral hemorrhage [Incidence Rate Ratio (IRR) 1.31] (22).

Atrial fibrillation (AF) increases stroke risk by greater than 5-fold (23), and it is independently associated with AD beyond the impact of stroke (24). A review by Ilhara & Washida cited three population-based cohort studies with a significantly increased AD hazard ratio (25). Although one study in the review did not show significant results, the authors hypothesized that this may be due to its restriction to participants 75 and older and that the impact of AF on AD risk may decline with age.

Having established these epidemiological associations between CVD and AD, we will now begin to unravel the pathways along the heart-brain axis by which these diseases may be related.

## Mechanistic pathways

**Figure 1:**
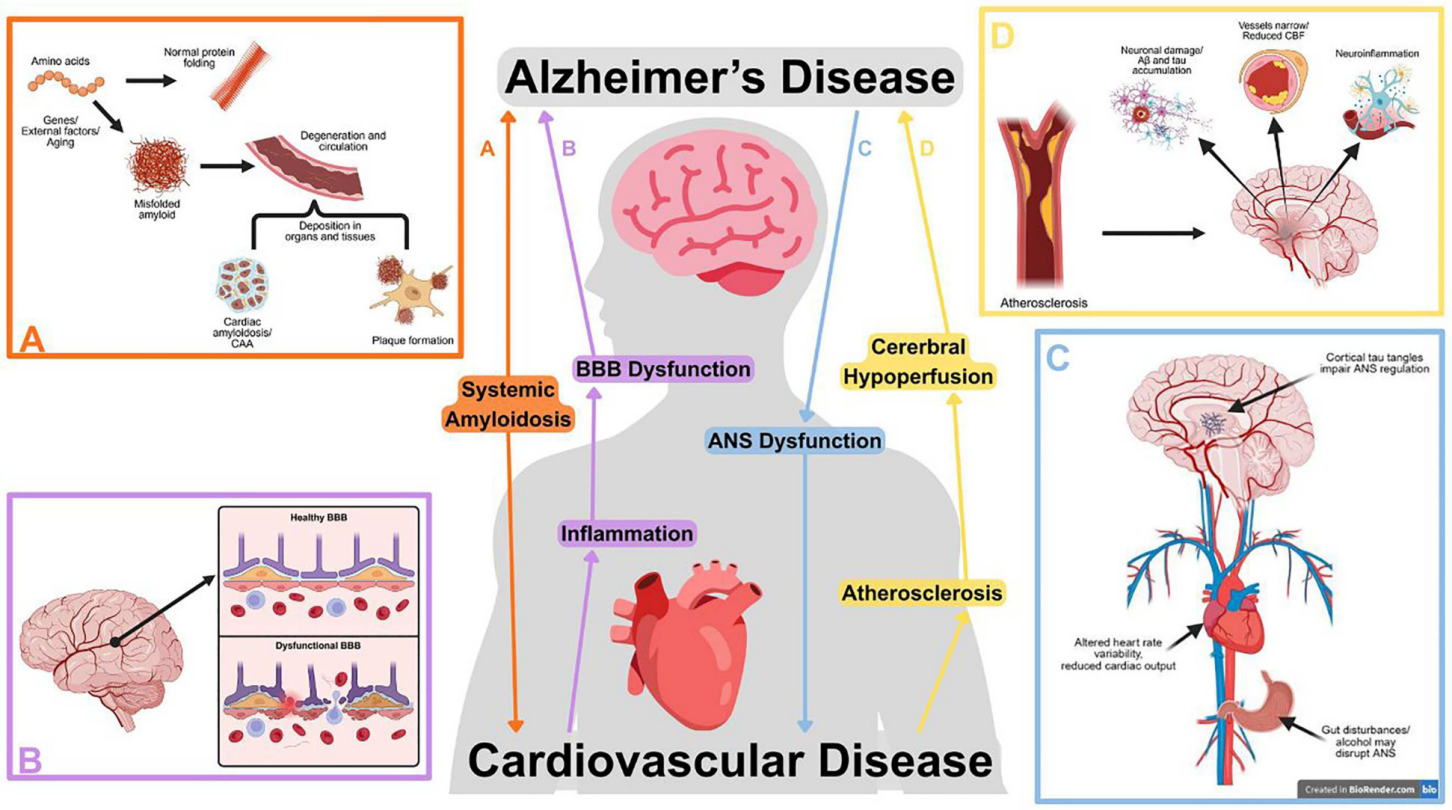
Selected pathways between cardiovascular and Alzheimer's disease (AD), illustrating the bidirectional relationship between cardiovascular dysfunction and neurodegeneration. Subfigures highlight pathways in further detail. **(A)** Systemically misfolded amyloid proteins can deposit in blood vessels, cardiac tissues, or form neural plaques. Created in BioRender. Zhou, W. (2025) https://BioRender.com/5n6b4oi. **(B)** Cardiovascular diseases can initiate a vicious cycle between inflammation and blood-brain barrier (BBB) breakdown, promoting the development of amyloid plaques and tau tangles. Created in BioRender. Zhou, W. (2025) https://BioRender.com/l54sgrk. **(C)** Dysregulation of the autonomic nervous system (ANS) disrupts cardiac function and may occur due to cortical tau tangles or outside factors such as alcohol consumption or gut disturbance. Created in BioRender. Zhou, W. (2025) https://BioRender.com/gzo4dey. **(D)** Atherosclerosis contributes to AD progression through reduced cerebral blood flow (CBF), neuroinflammation, and promoting amyloid and tau accumulation. Created in BioRender. Zhou, W. (2025) https://BioRender.com/0lcsbdv.

The heart-brain axis involves the complex interactions between the cardiovascular and central nervous systems, mediated through various physiological and biochemical mechanisms. Understanding these pathways is crucial for elucidating bidirectional communication and its implications for cardiovascular and cognitive health. Figure 1 displays the interconnected pathways of CVD and AD. Here, we review several critical mechanisms linking AD and CVD in the heart-brain axis.

### Cerebral hypoperfusion

A head-to-heart link has been hypothesized through the reduction of blood flow to the brain, or cerebral hypoperfusion. The brains receives 15% of cardiac output and is particularly sensitive to the effects of hypoperfusion (8). Cerebral hypoperfusion occurs due to three general mechanisms: vascular structural lesions from stenosis or artery occlusion, cerebral hemodynamic alterations, and changes in blood composition increasing viscosity (26). The variety of these mechanisms reflect the assorted CVD subtypes known to reduce cerebral blood flow such as atherosclerosis, arterial stiffness, AF, HF, and orthostatic hypotension (8, 24, 27). Atherosclerosis is an especially notable driver of hypoperfusion, as atherosclerotic plaques block blood flow and stiffen blood vessels. Increased pulse pressure damages cerebral microvasculature, disrupting oxygen delivery to the brain and impairing cognition (28, 29).

Cerebral blood flow (CBF) has a direct positive correlation with cognitive function (6). While the complex interplay between cardiovascular and neurodegenerative diseases makes causality difficult to determine, studies have shown that CBF reductions often precede brain atrophy in dementia, suggesting a contributing role to cognitive decline (30).

Several mechanisms have been proposed describing the pathways by which cerebral hypoperfusion disrupts brain homeostasis (31). Hypoperfuse regions of the brain receive insufficient oxygen, and the resulting mitochondrial dysfunction induces the development of AD pathology (26). Oxidative stress and corresponding inflammation upregulate amyloid precursor protein (APP) processing, which leads to elevated Aβ levels even in cases of mild cerebral hypoperfusion (26, 27). Hypoperfusion decreases glucose metabolism, accelerating the development of tau neurofibrillary tangles (26).

Cerebral hypoperfusion has also been found to contribute to AD pathology through cerebral damage independent of amyloid and tau pathways. Decreased CBF damages neurons, contributing to functional dysconnectivity in the early stages of AD (30). Chronic cerebral hypoperfusion leads to neuronal loss that directly correlates with dementia severity (26). Regions of cell death in MCI and AD are believed to be the result of hypoperfusion, contributing to atrophy in memory-associated lobes such as the hippocampus (32). The disrupted blood flow to the brain leads to structural changes contributing to cognitive impairment (6).

Most research focuses on cerebral hypoperfusion as a pathway from cardiac dysfunction to dementia, but some studies have investigated how AD pathology may contribute to reductions in CBF. Zhao and Gong reported that cerebrovascular lesions in animal models induced cerebral hypoperfusion (26). Additionally, Austin et al. described how low levels of soluble amyloid can lead to global impairment of vascular responses and AD-related dysregulation by impairing the action and synthesis of nitrous oxide (30). These findings suggest that cerebral hypoperfusion may be both a driver and consequence of AD pathology, reinforcing the bidirectional nature of the heart-brain axis.

### Inflammation and oxidative stress

Inflammation and oxidative stress play central roles linking CVD and AD. While it remains unclear whether neuroinflammation initiates AD, contributes to its pathology, or reflects a response to it (33), evidence suggests that Aβ accumulation around blood vessels may trigger vascular inflammation and cerebrovascular lesions that contribute to cognitive impairment (19, 34). Such lesions have been associated with neuroinflammation and subsequent cognitive decline, especially following coronary events (35). Myocardial infarction, for example, may induce systemic inflammation that accelerates neurodegenerative processes without directly causing classical AD pathology (36).

CVD-related inflammation involves both central and peripheral mechanisms. Pro-inflammatory cytokines are elevated following cardiac events and can activate neural pathways in the hypothalamus and brainstem, promoting neurogenic inflammation and BBB disruption (37). Activated platelets, when upregulated after acute coronary events, can adhere to cerebrovascular lesions and contribute to Aβ deposition in the brain (37). These events may be further compounded by age-related changes to inflammatory regulation. For example, cardiac dysfunction, increased arterial stiffness, and fluctuating blood pressure have been linked to microglial activation, tau phosphorylation, and hippocampal vulnerability (38).

Oxidative stress is another shared pathological mechanism. It is both a cause and consequence of Aβ and tau accumulation (39). The brain's high lipid content and metabolic demands make it especially susceptible to damage induced by reactive oxygen species (ROS), impairing synaptic function and promoting neurotoxicity. Free radicals generated during mitochondrial respiration, especially in the presence of unregulated metal ions, exacerbate Aβ toxicity and tau dysfunction (39). ROS in the brain can further affect the cardiovascular system, as they may disrupt neural signaling and influence systemic blood pressure through neuroendocrine and autonomic pathways (40). Oxidative and inflammatory changes have been observed in peripheral tissues and cardiomyocytes in AD patients, further supporting a systemic disease model (38, 41).

Chronic inflammation, often driven by metabolic comorbidities, is a common feature of both CVD and AD (42). Under neuropathological conditions, peripheral inflammatory signals can cross the BBB and trigger sustained activation of microglia and astrocytes, creating a cycle of synaptic damage, neurodegeneration, and worsening pathology surrounding Aβ plaques (42).

### Blood-brain barrier dysfunction

The BBB plays a critical role in the heart-brain axis by tightly regulating the exchange of molecules between circulating blood and the brain. Composed of endothelial cells, astrocytes, pericytes, and microglia, the BBB safeguards neural function through tight junctions and selective transport mechanisms (38, 43). The BBB plays an important role in AD pathology, as its integrity is essential for clearing Aβ from the brain and preventing its influx. When these mechanisms become impaired, the balance is tipped toward Aβ accumulation (38, 44). Importantly, BBB dysfunction is considered an early biomarker of cognitive decline, with damage often appearing in the hippocampus years before clinical symptoms emerge (44).

Disruption of BBB integrity contributes to neuroinflammation and impaired CBF, potentially initiating a vicious cycle of damage. Aβ itself can exert toxic effects on endothelial cells and pericytes, increasing permeability (38, 45). Harmful plasma components can then enter the brain, activating glial cells and exacerbating inflammation (38, 44). BBB breakdown is linked to the accumulation of toxic molecules, hypoxia, and tau pathology, all of which accelerate neurodegeneration (33, 44). Autopsy and biomarker evidence confirm widespread BBB disruption in AD patients (44).

The connection between CVD and BBB dysfunction is strong, highlighting its prominence as a link between pathologies of the heart and brain. Hypertension and chronic cerebral hypoperfusion damage the cerebrovascular structure, reduce cerebral blood flow, and disrupt the BBB's tight junctions (45, 46). BBB damage has been associated with cerebral small vessel disease, microbleeds, and metabolic changes, all of which contribute to cognitive decline in AD (43, 45). CVD-induced inflammation results in circulating cytokines and immune cells that infiltrate the compromised BBB, activating microglia and astrocytes, which in turn perpetuate cytokine release, endothelial damage, and oxidative stress (44–46). These effects are further compounded by the BBB's failure to eliminate neurotoxic metabolites and its impaired nutrient transport systems (45).

BBB disruption often parallels AD progression, and some studies suggest it may reflect cerebrovascular damage independent of amyloid or tau pathology, potentially indicating mixed pathology with vascular dementia (45). This has prompted interest in the BBB not only as a passive marker of neurodegeneration but also as an active component of the heart-brain axis. The complex interplay of CVD, inflammation, and BBB dysfunction underscores its potential as a therapeutic target for preventing or slowing cognitive decline in aging populations (44, 46).

### Autonomic dysfunction

The autonomic nervous system (ANS) plays a vital role in maintaining homeostasis and regulating cardiovascular and neural functions. In the context of the heart-brain axis, growing evidence suggests that dysfunction of the ANS, or dysautonomia, may be a significant mediator of AD progression and CVD risk (47, 48). AD patients frequently exhibit vascular autonomic disturbances such as orthostatic hypotension and altered heart rate variability, which can result in inadequate cerebral perfusion and exacerbate cognitive decline (47, 49). These autonomic disturbances may stem from tau pathology in cortical regions governing autonomic control or impaired neurovascular regulation through cholinergic neuron degeneration (47, 49).

Loss of cholinergic input to cerebral blood vessels disrupts vasodilation and contributes to Aβ accumulation, further damaging vascular integrity and amplifying neurodegeneration (49). Reduced parasympathetic activity and cholinergic dysfunction have been strongly correlated with the degree of cognitive impairment in AD, and it has been hypothesized that this may be mediated by reductions in CBF (49). However, clear causal evidence linking autonomic dysfunction with CBF regulation in AD remains limited, underscoring the need for further investigation (49).

The ANS also exerts a bidirectional influence on cardiac function signaling pathways. Dysregulation of adrenergic and cholinergic signaling has been shown to contribute to arrhythmias and exacerbate cardiac pathology in individuals with pre-existing cardiovascular conditions (48). Progressive impairment of neurotrophic signaling pathways may lead to degeneration of autonomic nerve fibers and increased CVD risk. However, some of these changes may reflect the effects of broader aging processes (48). Importantly, autonomic remodeling observed in CVD may impact the brain in return, affecting neuronal health and cognitive stability.

Common risk factors for AD and CVD have been associated with dysfunction of the ANS. Bruning et al. suggested that microbial gut disturbances may link CVD and neurodegenerative changes (50). Alcohol consumption can also disrupt the ANS, as ethanol and its byproduct acetic acid can lead to oxidative stress, subsequent changes in arterial pressure, and sympathetic nervous system activation (SNA) (51). This research suggests that preserving ANS integrity through mechanisms such as gut health and reducing alcohol use may improve health outcomes across the heart-brain axis.

### Systemic amyloidosis

Aβ accumulation in the brain has traditionally been viewed as the hallmark feature of AD. Now, researchers are beginning to question whether the systemic deposition of misfolded proteins throughout the body may drive the link between AD and CVD. Cerebral amyloid angiopathy (CAA), marked by the deposition of Aβ in the walls of cerebral vessels, is found in 80%–100% of cases of AD and contributes significantly to microvascular dysfunction, hypoperfusion, oxidative stress, and cognitive decline (38, 45). The progression of CAA is prompted by impaired amyloid clearance, particularly through compromised drainage pathways which themselves depend on healthy cardiac output (52, 53). Systemic amyloid dysregulation may underpin cortical atrophy in AD and CVD-related vessel damage (34, 54).

AD patients often exhibit signs of subclinical heart disease that may be linked to cardiac amyloid deposits, including aortic valve thickening, ventricular wall hypertrophy, and infiltrative cardiomyopathy (54). Some studies have found low concentrations of amyloid fragments in cardiac tissue of AD patients, but others have reported no direct correlation between cerebral and cardiac amyloid levels, raising questions about whether these deposits arise from common systemic mechanisms or independent processes (54, 55). Still, amyloid aggregates have been associated with platelet activation, plaque rupture, and thrombosis, suggesting the protein plays a pathogenic role in coronary artery disease (CAD) and HF (52).

Amyloid's role in the heart-brain axis is reinforced by the bidirectional nature of its effects. Circulating Aβ may contribute to multi-organ endothelial dysfunction and inflammation, leading to poor clinical outcomes (52). In the brain, vascular amyloid impairs BBB integrity (53, 56). Similarly, amyloidosis in the heart can lead to arrhythmias, impaired contractility, and autonomic dysfunction, which may exacerbate cerebral hypoperfusion and worsen AD pathology (34). Moreover, systemic amyloid toxicity induces oxidative stress, calcium dysregulation, and apoptosis, driving further degeneration and protein misfolding in the heart and brain (57).

Despite strong pathophysiological parallels, uncertainty remains regarding whether amyloidosis reflects a truly systemic condition that bridges CVD and AD or instead represents distinct but converging processes in each organ system. Some researchers argue that amyloid may follow similar misfolding and clearance pathways in both the brain and heart, but its deposition is independently triggered by local stressors, aging, or metabolic dysfunction (55, 57). Regardless, the shared inflammatory, oxidative, and vascular effects of amyloid pathology across the brain and cardiovascular system underscore its potential importance as a mechanistic role-player the heart-brain axis.

## Imaging-derived insights

Medical imaging technologies greatly enhanced our understanding of connections throughout the heart-brain axis. Here, we highlight the findings from MRI and radiotracer imaging studies. See Table 1 for a comprehensive list of imaging studies of the heart-brain axis and their major findings.

**Table 1:** Imaging studies of the heart-brain axis.

Section	Reference	Title	Year	Imaging type	Major findings
Structural links from multi-organ imaging	(67)	Heart-brain connections: Phenotypic and genetic insights from magnetic resonance images	2020	Cardiac MRI, Brain MRI, fMRI, DTI	Heart MRI traits associated with gray matter morphometry, white matter microstructure, and functional networks. Cardiac wall thickness associated with subcortical brain structure volumes, including putamen. White matter integrity associated with aortic areas and left ventricle traits. Resting functional MRI had negative correlation with chamber volumes. Somatomotor network positively associated with chamber volumes, global peak circumferential strain, and global wall thickness; negatively correlated to ejection fractions.
	(70)	A structural heart-brain axis mediates the association between cardiovascular risk and cognitive function	2024	Cardiac MRI, Brain structural MRI, DTI	Heart and brain structure covariance significantly mediated the association between vascular risk factors and cognitive function. Heart structural variation is associated with cognitive function in ways independent of brain structural variation.
	(71)	Machine-learning-derived heart and brain age are independently associated with cognition	2023	Brain MRI	Heart delta age and brain delta age were significantly correlated. Brain delta age mediated a small portion of the heart delta age effect on cognitive function.
CVD-related cortical atrophy and lesions	(72)	Volumetric brain MRI signatures of heart failure with preserved ejection fraction in the setting of dementia	2024	T1-weighted MRI	Identified six brain regions with significant atrophy associated with HF with preserved ejection fraction in dementia patients: accumbens area, amygdala, posterior insula, anterior orbital gyrus, angular gyrus, and cerebellar white matter.
	(76)	Brain imaging and cognitive predictors of stroke and Alzheimer disease in the framingham heart study	2013	Brain MRI	Hippocampal volume predicted AD. Vascular risk factors and MRI markers predicted stroke, not AD.
	(77)	Cardiometabolic disease, cognitive decline, and brain structure in middle and older age	2024	Brain MRI	Cardiometabolic diseases were associated with smaller brain volumes and more white matter hyperintensities starting in middle age, and association with cognitive decline emerges in older age.
	(74)	Clinical and imaging markers of cardiac function and brain health	2025	Echocardiography, cardiac MRI, brain MRI	Systolic and diastolic dysfunction as well as clinical heart failure were associated with smaller brain volumes.
	(75)	Cognitive deficits and related brain lesions in patients with chronic heart failure	2018	Brain MRI	HF patients had attention and memory deficits. Medial temporal lobe atrophy but not white matter lesion load was associated with severity of cognitive impairment.
	(147)	Heart rate, brain imaging biomarkers and cognitive impairment in older (≥63 years) Women	2020	Brain MRI	Elevated resting heart rate was associated with a greater brain lesion volume and a significant increase in the number of lesions between exams in postmenopausal women without a history of CVD, but neither was related to cognitive impairment.
	(9)	Dementia and Alzheimer's disease incidence in relationship to cardiovascular disease in the cardiovascular health study cohort	2005	Brain MRI	Older adults with non-stroke CVD had heightened risk of all-cause dementia and AD. The highest risk was for patients with peripheral artery disease.
	(138)	High cognitive reserve attenuates the risk of dementia associated with cardiometabolic diseases	2024	Brain MRI	Higher levels of cognitive reserve were associated with a lower risk of dementia and larger gray matter and hippocampal volumes in people with cardiometabolic disorders.
	(80)	Hyperintense brain lesions in asymptomatic low risk patients with paroxysmal atrial fibrillation undergoing radiofrequency pulmonary vein isolation	2021	Brain MRI	More white matter hyperintensity lesions were found in older patients with a higher cardiovascular risk score. Cerebral microembolism was not related to cognitive impairment in this population.
	(78)	Mixed brain lesions mediate the association between cardiovascular risk burden and cognitive decline in old age: A population-based study	2016	Brain MRI	A higher cardiac risk score was associated with faster cognitive decline in individuals 60-72 years old. Mixed brain lesions worked as mediators that substantially attenuated the association between cardiac risk score and cognitive decline.
	(148)	Prolonged ventricular repolarization associated with mild cognitive impairment and white matter hyperintensities: a cross-sectional study	2024	Brain MRI	Prolonged ventricular repolarization was associated with MCI and cerebral microvascular lesions in a general population of older adults.
	(133)	Sex differences in cognitive functioning in patients with heart failure	2025	Brain MRI	Women with HF performed better in global cognition than men, which was explained by a predominance of preserved left ventricular ejection fraction as opposed to ischemic heart failure. Adjustment for non-lacunar infarcts explained memory differences between sexes.
	(73)	A population-based study on blood pressure and brain atrophy in 85-year-olds	1998	CT	Systolic and diastolic blood pressure were negatively correlated with dementia severity. Frontal cortical atrophy was correlated to lower systolic blood pressure.
	(81)	Association between vascular comorbidity and progression of Alzheimer’s disease: a two-year observational study in Norwegian memory clinics	2018	Brain MRI	No association found between vascular risk factors and diseases, Framingham Stroke Risk Profile, nor cerebrovascular disease and AD progression over 2 years of follow up.
	(79)	Blood-brain barrier integrity disruption is associated with both chronic vascular risk factors and white matter hyperintensities	2025	Brain MRI	Higher blood pressure and body mass index were associated with greater BBB disruption. BBB disruption, as assessed with a Transendothelial Electrical Resistance assay, was associated with a higher white matter hyperintensity burden.
Arterial calcification and cognitive function	(82)	Arterial calcification in the heart–brain axis and cognitive performance over time	2024	Noncontrast CT	High arterial calcification was associated with worse baseline cognitive performance. Arterial calcification closest to the brain had greatest impact. Relationships may be mediated by chronic hypoperfusion and small vessel disease.
	(83)	Association of arterial structure and function with incident cardiovascular diseases and cognitive decline	2025	Carotid ultrasound, brain MRI	CIMT had robust association with white matter hyperintensity, incident CVD, and dementia.
	(87)	Association of annular calcification and aortic valve sclerosis with brain findings on magnetic resonance imaging in community dwelling older adults: the cardiovascular health study	2011	Brain MRI, echocardiography	Presence of valve calcification was associated with a higher prevalence of MRI-defined brain infarcts. Associations remained significant after adjusting for covariates.
	(86)	Carotid atherosclerosis promotes the progression of Alzheimer's disease: A three-year prospective study	2017	Carotid Ultrasound	CIMT was significantly associated with measures of cognitive function. Greater CIMT values were associated with faster cognitive decline.
	(84)	Coronary Artery Calcium, Brain Function and Structure: The AGES-Reykjavik Study	2010	CT, Brain MRI	Increasing coronary artery calcification was associated with poorer cognitive performance and dementia, which may be mediated by brain lesions and volumes.
	(85)	Coronary artery calcium: associations with brain magnetic resonance imaging abnormalities and cognitive status	2005	Electronic beam tomography, Brain MRI	Older adults with higher coronary artery calcification were more likely to have more severe subcortical infarction and white matter hyperintensities that attenuated the association between coronary artery calcification and abnormal cognitive status.
AD proteinopathies	(91)	18FFlutemetamol-PET aided classification of cerebral amyloid angiopathy	2024	18FFlutemetamol-PET	In group of patients with intracranial hemorrhage, CAA/amyloid pathology-predominant have higher lobar microbleed burden than atherosclerosis-predominant participants. CAA/amyloid pathology group also had higher rate of adverse outcomes in follow up.
	(88)	Association of β-amyloid and vascular risk on longitudinal patterns of brain atrophy	2022	PiB-PET	High vascular risk and elevated Aβ burden interacted to predict more longitudinal brain atrophy, which influenced cognitive decline.
	(92)	Age, vascular health, and Alzheimer disease biomarkers in an elderly sample	2017	Amyloid PET, tau PET, MRI, subset with FDG PET	Presence of cardiometabolic conditions associated with greater neurodegeneration. No direct association between poor vascular health and amyloid deposits. Significant impact of vascular health on tau driven by neurodegeneration.
	(95)	Association of mid-life cardiovascular risk with biomarkers of Alzheimer's disease, neurodegeneration, and white matter hyperintensities: Heart SCORE brain study	2025	Brain MRI, PiB-PET, Flortaucipir PET	Atherosclerotic CVD risk was associated with late-life neurodegeneration and white matter hyperintensities, not amyloid or tau.
	(90)	Associations between atrial arrhythmias and brain amyloid deposition: The ARIC-PET study	2022	FBP-PET	No association between atrial arrhythmias and Aβ burden in nondemented community-dwelling older adults.
	(149)	Associations between atrial cardiopathy and cerebral amyloid: The ARIC-PET study	2020	FBP-PET, echocardiogram	Significant association between atrial cardiopathy, left atrial volume index, and elevated brain amyloid compared to controls without AF.
	(96)	Cerebral hypoperfusion is not associated with an increase in amyloid β pathology in middle-aged or elderly people	2017	18F-Flutemetamol PET, Subset with 18F-AV-1451	Reduced CBF found in patients with arterial occlusion/stenosis, but no association with tracer uptake. Suggests cerebral hypoperfusion is not a causative event in amyloid accumulation.
	(150)	Blood-brain barrier disruption and perivascular beta-amyloid accumulation in the brain of aged rats with spontaneous hypertension: evaluation with dynamic contrast-enhanced magnetic resonance imaging	2018	DCE-MRI	BBB disruption was associated with Aβ influx and accumulation in brains of aged rats with hypertension.
	(89)	Do cardiometabolic risk factors influence amyloid, tau, and neuronal function in APOE4 carriers and non-carriers in Alzheimer’s disease trajectory?	2018	AV45-PET, 18F-FDG-PET, MRI	AD-related neuropathological changes were accelerated in association with vascular risk profiles in both APOE4 carriers and non-carriers.
	(93)	Tau pathology and relative cerebral blood flow are independently associated with cognition in Alzheimer’s disease	2020	18F-flortaucipir PET	Tau pathology was associated with reduced relative CBF, and both were independently associated with worse cognitive performance, indicating they may contribute separately to cognitive impairment in AD.
	(94)	Tau pathology as determinant of changes in atrophy and cerebral blood flow: a multi-modal longitudinal imaging study	2023	18F-flortaucipir PET, brain MRI	Higher tau load was related to accelerated cortical thinning but not decreases in relative CBF.
Glymphatic dysfunction	(141)	Ablation and pacing: improving brain perfusion and cognitive function in patients with atrial fibrillation and uncontrolled ventricular rates	2011	99mTc-HMPAO SPECT	Patients with AF have lower CBF than healthy controls. Ablation and pacemaker implementation improved blood pressure and cognitive function.
	(100)	Association of glymphatic system dysfunction with cardiac injury and cognitive impairment in heart failure: A multimodal MRI study	2025	Brain MRI, cardiac MRI	Patients with heart failure have glymphatic dysfunction, with mean diffusivity along perivascular spaced closely correlated to cardiac function and cognition. Glymphatic dysfunction in HF patients may lead to cognitive impairment.
	(99)	Associations of blood cardiovascular biomarkers with brain free water and its relationship to cognitive decline	2023	Diffusion MRI	Brain free water mediated relationship between cardiovascular blood biomarkers and cognitive decline.
	(101)	Associations of ischemic heart disease with brain glymphatic MRI indices and risk of Alzheimer's disease	2025	Brain MRI, DTI	Ischemic heart disease is independently associated with AD risk, and free water markers indicate that brain glymphatic dysfunction may partially mediate this relationship.
Inflammation and nervous system dysfunction	(102)	Imaging the molecular footprints of the heart–brain axis in cardiovascular disease	2019	TSPO PET, 11C-PK11195 PET	Myocardial infarction leads to systemic inflammation, including neuroinflammation. TSPO PET signal showed biphasic inflammation in both heart and brain in mouse models.
	(104)	Association between inflammatory biomarkers and cognitive aging	2022	Brain MRI	Higher circulating levels of certain inflammatory proteins associated with poorer performance on neuropsychological tests. Higher CD5L was associated with smaller total brain volumes and higher circulating soluble RAGE was associated with larger total brain volume.
	(103)	Neuroinflammation is independently associated with brain network dysfunction in Alzheimer’s disease	2022	11C-PBR28 PET, 18F-flutemetamol PET, T1-MRI, DTI, rsfMRI	Cortical neuroinflammation coincides with structural and functional network disruption independent of Aβ and cortical atrophy. Pathway is suggested from neuroinflammation to systemic brain dysfunction in AD.
	(105)	Imaging of the brain–heart axis: prognostic value in a European setting	2024	FDG-PET/CT	SNA was a robust and independent predictor for all-cause mortality, but association between SNA activation and major adverse cardiac events was lost when adjusting for cardiovascular status.

Two major imaging modalities have been implemented to understand the heart-brain axis: magnetic resonance imaging (MRI) and radiotracer imaging. MRI is a powerful non-invasive imaging technique that uses strong magnetic fields and radiofrequency pulses to produce high-resolution images of internal body structures (58). It is widely used in clinical and research settings due to its safety, accessibility, and ability to generate detailed anatomical and functional information. However, MRI can be uncomfortable for patients, and its high cost may limit availability (7, 32). In recent years, multi-organ MRI has revolutionized our understanding of the heart-brain axis by allowing simultaneous imaging of cardiac and cerebral structures.

Nuclear medicine, in the form of single photon emission computed tomography (SPECT) and positron emission tomography (PET) is another imaging technology utilized to understand the heart-brain axis. Both techniques utilize radioactive tracers administered to the patient, but SPECT sensors detect gamma rays, while PET scans track positrons released by the tracer (59). SPECT has greater availability than PET, while PET offers better resolution and more sophisticated tracers (60). Additionally, while PET cannot localize brain activity as precisely as MRI, tracers can be used to highlight deposition of certain compounds throughout the body, such as Aβ and neurofibrillary tau in AD (7).

### Structural links from multi-organ imaging

Recent findings from imaging studies have revolutionized our understanding of the intricate relationship between the heart and the brain (61). These innovations have enabled more precise and comprehensive investigations into how cardiovascular conditions influence brain health and function. The combination of cardiac and brain MRI in a single session is a growing field in imaging. For example, multi-organ MRI scans now provide a comprehensive view of both the heart and brain in a single setting, helping to identify structural changes associated with CVD and cognitive decline **(**62). This approach is particularly valuable in clinical settings, as both conditions often coexist.

In 2023, Zhao et al. published a groundbreaking study regarding the heart-brain axis (63). They found structural and functional correlations between heart and brain health, highlighting correlated structures derived from multimodal MRI. For instance, cardiac wall thickness traits were linked to the volumes of subcortical structures related to cognitive function (63). This association may be mediated by systemic hypoperfusion (64). Cardiac chamber volumes had a negative correlation with resting state functional MRI (63), which may be explained by shared cardiac risk factors, such as atherosclerosis, contributing to brain dysfunction or ventricular enlargement as a compensatory mechanism for perfusion deficiencies (65). Our understanding of the heart-brain axis is greatly enhanced by large-scale studies providing such insight.

Another study by Jaggi et al. (66) explored the heart-brain axis using covariance of cardiac and brain MRI features. Three key components each were retained to convey the information of heart and brain MRI. The study identified significant correlations between heart and brain components. Notably, individuals with four or more vascular risk factors demonstrated reduced left ventricular volumes, lower myocardial intensity, and significant grey matter loss, which correlated with lower cognitive function scores. The study found that the component connecting myocardial intensity, grey matter volume, and thalamic white matter integrity fully mediated the relationship between vascular risk and cognitive function. However, brain structural variation did not mediate the relationship between heart variation and cognition, indicating independent effects of heart and brain structure on cognitive decline. The independent roles of heart and brain structure on cognitive function are supported by the work of Iakunchykova et al. (67). A 2023 study examined the differences between subjects’ chronological ages and machine-learning-derived heart and brain age estimates (delta ages) based on electrocardiogram and brain MRI, respectively. A significant correlation was found between brain and heart delta ages, underscoring the linked health of these two organs. Importantly, brain delta age only mediated 13% of the association between heart delta age and cognitive scores. Together, these studies highlight the separate importance of heart and brain structural integrity on cognitive function.

### CVD-related cortical atrophy and lesions

Many neuroimaging studies have examined the presence of cortical atrophy and lesions in CVD patients, and the respective relationship between brain deterioration and clinical dementia. The concept of the heart-brain axis is strengthened by findings of cortical atrophy in CVD patients. Bermudez et al. (68) leveraged high-resolution brain MRI to identify specific regions of the brain, such as the accumbens area, amygdala, and posterior insula, showing atrophy in patients with both dementia and heart failure with preserved ejection fraction. Blood pressure has long been associated with cortical atrophy, as a 1998 study by Skoog et al. found systolic and diastolic blood pressure to be negatively correlated with dementia severity and frontal cortical atrophy to be correlated to lower diastolic blood pressure (69). The finding of reduced blood pressure in dementia patients may occur from AD-induced ANS dysfunction. Alternatively, lower blood pressure may cause cortical atrophy via cerebral hypoperfusion. Recently, Yaqub et al. performed a meta-analysis of studies discussing cardiac function and structural markers on brain MRI and found robust evidence suggesting an association between systolic and diastolic dysfunction and smaller total brain and hippocampal volumes in cardiac patients (70). Frey et al. found atrophy severity in the medial temporal lobe, which contains memory-related structures such as the hippocampus and amygdala, to be associated with the degree of cognitive decline in chronic HF patients (71). Low hippocampal volume has been found to predict AD in addition to general cognitive decline (72). The combined weight of these studies suggests a link from CVD to cognitive decline via brain atrophy, particularly in the medial temporal lobe.

Another form of neurodegeneration associated with CVD and cognitive decline is the presence of cortical lesions. These lesions are identified as white matter hyperintensities (WMH) on structural brain MRI. In a UK Biobank study, Dove et al. identified a relationship between a high number of cardiometabolic diseases (CMDs) and WMH volume in middle and older age (73). They also found a correlation between the number of CMDs and rate of global cognitive decline in older age, suggesting that CMDs contribute to neurodegeneration, which results in cognitive decline later in life. Evidence from Wang et al. supports this pathway, as they found mixed brain lesion burden to largely mediate the correlation between cardiac risk score and cognitive deterioration in participants aged 60–72 (74). BBB dysfunction may be a key link between cardiac risk factors and cognitive impairment, as BBB integrity was associated with cortical damage and cardiac risk factors such as high body mass index (BMI), systolic and diastolic blood pressure, elevated blood glucose, and lipid levels (75). Several specific forms of cardiac dysfunction have been associated with brain lesions. For example, Mao et al. found prolonged ventricular repolarization to be related to total WMH burden and MCI in the general population of older adults. In a study of AF patients, more WMHs were found in older patients and those with a higher cardiovascular risk scores (76).

Although some studies have identified a correlation between white matter lesions and cognitive function, others have not. In the AF population undergoing radiofrequency pulmonary vein isolation, Wieczorek et al. did not note a relationship between cerebral microembolism and cognitive decline (76). This may be due to the relatively young population (median 58.5 years), as the cognitive effects of white matter lesions might not emerge until later in life. Another study by Eldholm et al. found no effect of vascular risk factors and MRI-detected cerebrovascular disease on AD progression over two years (77). The authors hypothesized that the follow-up time may have been insufficient to note changes. These findings suggest that white matter lesions may only link the heart to cognitive functions at certain times of life or in certain populations and reinforce the importance of examining these effects in varying populations.

### Arterial calcification and cognitive function

Arterial calcification plays an important role in the heart-brain axis, and its function is supported by imaging evidence. High arterial calcification, as assessed by noncontrast computed tomography (CT) scan is associated with poorer baseline cognitive performance, especially in the arteries closest to the brain (78). This relationship is thought to be mediated by chronic hypoperfusion and small vessel disease. Other studies support the contributing role of arterial calcification in cerebral small vessel disease, as Robert et al. found carotid intima-media thickness (CIMT) identified through carotid ultrasound to be robustly associated with WMHs, incident CVD, and dementia (79). Additionally, data from the AGES Reykjavik Study showed that subjects with high coronary artery calcification were more likely to have lower white and gray matter volumes and more brain lesions, which significantly attenuated the association between atherosclerotic burden and dementia (80). This supported similar findings in a 2005 cross-sectional study in which electron beam tomography was used to detect coronary artery calcification (81). A prospective study of AD patients found higher CIMT values to predict faster cognitive decline, indicating that arterial thickness not only increases risk for dementia, but also impacts its progression (82). Other forms of cardiovascular calcification have been found to be associated with brain health as well. Rodriguez et al. found both annular and valvular calcification to be associated with covert brain infarcts, which can lead to reduced CBF and consequent cognitive decline (83).

### AD proteinopathies

Imaging studies exploring the heart-brain axis have utilized PET tracers to understand the impacts of cardiovascular factors on Aβ and tau accumulation patterns. Radiotracer imaging of AD-specific proteinopathies offers an important perspective for understanding the separate effects of vascular and AD dementia.

In a study of adults from the Harvard Aging Brain Study, Rabin et al. found that vascular risk, according to the Framingham Heart Study cardiovascular disease risk score, and Aβ burden, quantified by Pittsburgh compound B (PiB) PET, interacted to predict more severe pathology in the frontal and temporal lobes, thalamus, and striatum. Damage to these regions in turn influences cognitive decline (84). Femminella et al. assessed the relationship between the Qrisk2 cardiovascular risk score and cerebral spinal fluid Aβ and tau, PET amyloid and metabolic imaging, brain MRI, and cognitive measures in APOE4 carriers and non-carriers (85). They found high cardiovascular risk to be associated with higher AD biomarker levels regardless of APOE4 status, indicating that control of modifiable cardiovascular risk factors could be an effective strategy to reduce AD risk. Specific forms of cardiac dysfunction have been tied to Aβ deposition, as there was a significant association between atrial cardiopathy, left atrial volume index, and elevated global cortical Aβ compared to controls without AF (86). Romoli et al. utilized 18Fflutemetamol PET to examine the differences between patients with arterial stiffening due to CAA amyloid pathology and predominant atherosclerosis (87). They found a higher lobar microbleed burden and rate of intracranial hemorrhage in the CAA predominant group, indicating that amyloid may have a greater impact on brain lesions than atherosclerosis. These studies highlight the complex interactions between cardiovascular function and Aβ accumulation in AD.

Imaging studies have also suggested a correlation between cardiovascular health and tau pathology. Vemuri et al. identified a strong association between vascular health and neurodegeneration in adults older than 60, with a weaker association between vascular health and tau deposition driven by hyperlipidemia, as cholesterol accumulation is toxic to neuronal cells and may be involved in tau generation (88). Amyloid accumulation, which was not associated with vascular health in this population, was the primary factor associated with tau accumulation. Other tau PET studies have investigated connections with CBF in AD patients. Visser et al. found tau pathology to be associated with locally reduced relative CBF (89). Both mechanisms were associated with worse cognitive performance, albeit through different pathways. Tau pathology affected the widespread neocortex, but relative CBF associations were restricted to lateral temporal and parietal regions. The authors suggested that these findings may show that tau pathology leads to decreased relative CBF, but a time lag may exist before changes in CBF emerge. Another study by Visser et al. examined the longitudinal relationship between tau and relative CBF, and they found that higher tau load was associated with accelerated cortical thinning, but not relative CBF (90). The lack of association between tau and CBF in this study may be due to the aforementioned lag effect in this relationship, and the two-year follow-up window of the study may have been insufficient to notice tau-driven changes in CBF.

However, other studies have not found a link between cardiovascular risk and AD-specific neuropathology. In the Heart SCORE Brain Study, mid-life atherosclerotic cardiovascular disease (ASCVD) risk was associated with neurodegeneration and WMH over approximately 16 years of follow-up, but not PET measurements of Aβ and tau (91). The lack of a relationship with these pathologic markers and ASCVD may be because the cohort was cognitively healthy, and that a small proportion of the participants were positive for Aβ and tau. Another study assessing patients with unilateral occlusion of the internal carotid artery or stenosis of the middle cerebral artery identified reduced CBF in the affected side of the brain, but PET imaging did not show associated deposits of Aβ or tau (92). Although this indicates that cerebral hypoperfusion due to large vessel occlusion may not induce AD pathology, the authors recognized that the effects of hypoperfusion due to cerebral small vessel disease may still play a contributing role. Uncertain past findings highlight the necessity of future research to untangle the details of the relationship between CVD and AD pathology.

### Glymphatic dysfunction

Diffusion tensor imaging (DTI) is an MRI technology that highlights the orientation of water molecules, and this picture of fluid movement through the brain enhances our knowledge of its functional networks (93). DTI is particularly useful in understanding the glymphatic system, the mechanism by which cerebrospinal fluid clears waste, including Aβ, from the brain (94). Ji et al. noted an association between cardiovascular-related blood biomarkers and brain free water and cognitive decline. Brain free water includes all water in cerebrospinal fluid and thus reflects glymphatic function. Free water fully mediated the relationship between the cardiovascular biomarkers and longitudinal cognitive decline over five years (95).

Other studies have utilized DTI to explore the role of glymphatic function in cognitive performance in patients with clinical CVD. Zheng et al. found heightened markers of glymphatic dysfunction in HF patients compared to matched controls (96). They further found that the mean diffusivity along perivascular space index mediated the correlation between stroke volume and cognitive testing results. A similar study by Wang et al. found evidence of glymphatic dysfunction in individuals with ischemic heart disease, which partially mediated the relationship between ischemic heart disease and AD risk (97). Cardiac dysfunction may damage the glymphatic system through mechanisms such as atherosclerosis and BBB dysfunction (94).

### Inflammation and nervous system dysfunction

Several imaging studies have examined the role of inflammatory and nervous system responses in the heart-brain axis. A significant addition to the understanding of the heart-brain axis came from a study by Thackeray et al. highlighting the molecular pathways involved in the reciprocal interaction between the heart and brain, particularly after ischemic cardiac injury (98). The study utilized mouse models to specifically examine how myocardial ischemia results in neuroinflammation, a key factor in cognitive impairment. Using advanced molecular imaging techniques such as PET imaging to track mitochondrial translocator protein as a marker of activated macrophages and microglia, the study revealed a biphasic neuroinflammatory response. Following myocardial infarction, there was an initial increase in neuroinflammation, followed by a decline, and then a second wave of inflammation several weeks later like the progression observed in AD pathogenesis. This response correlates with decline in cardiac function, reinforcing the idea that heart injury and the subsequent inflammatory cascade can impair brain health.

The role of neuroinflammation in cognitive decline in humans was observed by Leng et al., who utilized multimodal brain imaging including 11C-PBR28 PET to quantify microglial activation, 18F-flutemetamol PET for Aβ deposition, and T1-weighted, diffusion tensor, and resting-state functional MRI to assess structural and functional networks (99). They found cortical 11C-PBR28 uptake to be correlated negatively with structural integrity and network local efficiency independently of Aβ deposition and cortical thickness, suggesting neuroinflammation has an independent effect on systemic brain dysfunction.

Fang et al. examined the association between plasma proteins related to immune and inflammatory responses, brain structure, and cognitive function (100). High circulating levels of soluble CD40L and myeloperoxidase, markers of immune activation, were associated with poorer performance on neuropsychological tests. CD5L, a protein involved in macrophage biology, was associated with smaller total brain volume, indicating immune activation may either contribute to or be a result of dementia pathology. Levels of sRAGE, a protein that may play an anti-inflammatory role, were associated with larger total brain volumes, suggesting that inflammatory control may offer protection against neurodegeneration.

Mental stress can also activate inflammation that contributes to CVD. In a European cohort of cardiovascular patients, stress-related neural activity, as assessed through 18F-FDG-PET scans, was found to be a significant predictor of all-cause mortality but not of major adverse cardiovascular events (MACE) (101). This study observed that higher SNA, particularly in the left amygdala to ventromedial prefrontal cortex ratio, was associated with a 2.5-fold increased risk of all-cause mortality. In contrast, while a 1.5-fold increased risk for MACE was noted in patients with high SNA, this association was not statistically significant after adjusting for cardiovascular status. 18F-FDG uptake in brain regions like the amygdala and prefrontal cortex increases during acute stress in patients with CAD. Interestingly, higher uptake in these brain areas correlates with increased systemic inflammation and myocardial FDG uptake, suggesting a vicious cycle where stress-induced neuroinflammation and cardiac injury mutually exacerbate each other (102).

## Common genetic signatures

The genetic basis underlying CVD and dementia has been a subject of increasing interest due to the observed epidemiological overlap between the two conditions. Understanding how genetic variants contribute to these diseases is essential for identifying potential therapeutic targets and intervention strategies.

Several cohort-based studies have examined whether cardiovascular health (CVH) and inherited risk jointly shape dementia incidence. Peloso et al. examined how CVH interacts with genetic risk in determining dementia risk, using a genetic risk score (GRS) based on common variants associated with dementia, and did not observe a significant interaction, suggesting that CVH and genetic factors independently contribute to dementia risk (103). In line with this, the polygenic risk score (PRS) for CAD and lifestyle factors showed additive, not synergistic, contributions to AD risk  (104). Lenhoff et al. extended these findings, reporting that a high cardiovascular PRS correlated with brain atrophy and white-matter lesions but not CBF, indicating that genetic risk for CVD likely influences brain health through other mechanisms (105).

A study on Swedish twins also explored the effect of a CAD GRS on dementia risk after a CVD diagnosis, finding high GRS to partially modify the relationship between CVD and dementia, particularly within the first three years after CVD diagnosis (106). This suggests that individuals genetically predisposed to CAD may have been more vulnerable to developing dementia after a cardiac event, even if CAD-related genes did not directly affect dementia risk. Interestingly, while there was no genetic overlap between CAD and AD, both conditions shared common genetic variants related to lipid metabolism, suggesting that lipid dysregulation may be a key pathway linking CVD and dementia. These findings suggest that genetic susceptibility to CAD may influence dementia risk indirectly through lipid-related pathways.

Mendelian randomization (MR) further supports causality. Siedlinski et al. demonstrated that genetically elevated blood pressure leads to disruption of cerebral white-matter tracts, although total WMH burden did not translate into global cognitive decline (107). Phenome-wide association studies (PheWASs) showed a genetic relationship between AD and cardiac risk factors such as BMI, metabolic rate, and lipid-related disorders. A later MR study showed these traits to be causally linked (108). A two-sample MR performed by Grace et al. linked CAD genetic risk to slightly elevated late-onset AD risk, but the effect disappeared after excluding APOE variants, suggesting that APOE might the primary contributor to shared causal effects for AD and CVD (109).

Among single genes, APOE dominates. The APOE4 allele is the most significant genetic risk factor for AD, and its association with CVD is well-documented. The APOE4 allele has been linked to an increased risk of both CVD and dementia, whereas the APOE2 allele appears to have a protective effect on dementia despite predisposing to certain forms of CVD (110). APOE's influence on lipid transport, BBB integrity, and immune modulation makes it a critical component of the heart-brain axis (111, 112). Consequently, the gene remains the principal locus scrutinized in both CVD and AD genetics  (113).

Several loci beyond APOE merit attention as well. Pathogenic mutations in PSEN1 and PSEN2 drive early-onset AD by increasing Aβ production and have also been had a relationship detected to idiopathic dilated cardiomyopathy and heart failure, implying a systemic amyloidosis phenotype (114). Network-based analyses nominate CPBP1 as an upstream regulator in both atherosclerosis and AD   (115). Colocalization studies highlight shared causal variants such as LPIN3 as well as AKR1A1, ANLN, and WNT7B in dementia and atherosclerosis (116). Vascular endothelial growth factor (VEGF), a master regulator of angiogenesis, likewise links CVD and dementia, reinforcing cerebral perfusion as a unifying pathway (117).

Genes modulating inflammation further knit the heart and brain together. The PLEC gene, for example, is upregulated in the left ventricular endothelium and cardiomyocytes in HF and brain astrocytes in AD (118). The expression of SETDB2 was found to play a regulatory role in anti-inflammatory responses, while SYK mediated microglial activation, neurotoxicity, and the immune response (115). Moreover, genes relating to inflammation may help explain the frequent overlap between AD and vascular dementia, as Zheng et al. identified 74 co-expressed genes and 8 pivotal diagnostic genes linking the two forms of dementia through mechanisms such as signal transduction, neuroinflammation, and autophagy (119).

Most genetic studies use clinical diagnoses as endpoints, but imaging-derived phenotypes offer deeper mechanistic insight. Zhao et al. found that cardiac MRI traits share causal variants with functional connectivity and white-matter microstructure; for instance, a locus at 15q21.1 links ascending-aorta area to networks governing cognition and emotion  (63). Such work illuminates how common genetics simultaneously sculpt cardiac structure and brain integrity, advancing our understanding of the heart-brain axis.

## Demographic interactions

Both age and sex modulate the heart–brain axis, shaping how CVD influences the onset and progression of AD and other dementias. Older age is the strongest demographic risk factor for CVD and AD alike, while biological and social differences between men and women modify how cardiovascular pathology translates into cognitive decline.

### Age

Age is an important factor to consider when discussing the heart-brain axis, as the relationship between heart and brain health changes over the course of a lifetime. Important patterns have been observed in the particularly detrimental effects of the emergence of poor CVH in midlife, likely reflecting cumulative cognitive effects of prolonged CVD. The impact of CVH on later cognition is highly age-dependent. Poor CVH in midlife consistently predicts the greatest dementia risk, whereas CVD that first manifests in late life is less impactful. For example, Liang et al. showed that every 10-year earlier onset of CHD raised the hazard of all-cause dementia, AD, and vascular dementia by approximately 25 % (120). Similarly, van Gennip et al. noted that midlife onset of CVD (before age 60) significantly increases dementia risk. Onset later in life does not show the same association, emphasizing midlife as a critical period where CVD onset poses the most substantial risk to future cognitive health (121). In a related study, Sabia et al. used the Life's Simple 7 cardiovascular health score in midlife to predict dementia risk and found that adherence to ideal CVH metrics at age 50 correlated with lower dementia incidence over a 25-year follow-up (122). Although this study did not compare the long-term effects of poor CVH assessed at other ages, it provides supporting evidence that maintaining optimal cardiovascular health in midlife may be protective against later-life dementia.

Several specific cardiovascular risk factors appear to have a greater effect on dementia risk in midlife. Hypertension has a particularly notable relationship with cognitive decline, as blood pressure is a risk factor for AD in midlife and early old age, but may confer a protective effect late in life (123). Interestingly, higher diastolic blood pressure at age 70 is associated with AD, while at age 75 it is more strongly related to vascular dementia, suggesting that high blood pressure earlier in life may be more likely to activate Aβ and tau pathology. Protectively, the strongest association between good systolic function and healthy brain volume was found to occur from age 40 to 70 (70). The association between BMI and AD behaves similarly. High BMI at younger or middle ages is a risk factor for dementia but has a protective effect at older ages (124). This may occur due to dietary changes in subjects with subclinical or clinical dementia, as cognitive decline may result in missed meals or insufficient nutrition. One study examine the lifetime association between AD GRS and BMI, and found that BMI trajectories diverge based on GRS at approximately age 50, suggesting that AD pathology may begin to affect eating patterns or metabolism at that point of life (122).

Collectively, these data point to a window, beginning in midlife, in which CVD and its risk factors accelerate brain aging through sustained atherosclerosis, cerebral hypoperfusion, and related mechanisms. It is highly important to recognize the interaction between age and the heart-brain axis, as the success of therapeutic interventions may vary by age. In a systematic review of 165 clinical trials for AD, it was found that only 8% of trial participants were 85 years or older, while patients under 80, who represent a minority of AD cases, accounted for 78% of the sample (125). Future studies should implement measures to contain subjects more representative of the population with dementia to better understand efficacy among the most affected age groups.

### Sex

Epidemiological evidence suggests that CVD affects dementia risk differently in men and women. Significant hazard ratios between CVH and dementia onset are higher and occur for longer in women, while age interacts more strongly with cardiovascular risk in men (126). Women with CVD are 1.5 times more likely than men to develop AD, but men exhibit a higher risk of vascular dementia in the context of heart failure, suggesting that the pathways through which CVH impacts the brain may differ between sexes (127). These associations vary for specific forms of CVD, as females had a steeper decline in executive function, but a smaller change in cognitive decline following myocardial infarction than men (128). The study's authors suggest that this difference may reflect sociological effects of the measures themselves rather than biological differences between sexes. In another study, women with HF performed better in global cognition testing than men, which was explained by the predominance of preserved left ventricular ejection fraction as opposed to ischemic heart failure (129). Adjusting for non-lacunar infarcts mediated memory differences between sexes, indicating that major brain blockages are more likely to affect cognition in men.

The differences in risk between men and women can be attributed to several factors, including sex-specific variations in the prevalence and control of cardiovascular risk factors such as hypertension, diabetes, and obesity. Women, for example, are more likely to have poorly controlled CVH, which may contribute to their higher susceptibility to dementia (126). Biological factors contribute to sex-based differences in the heart-brain axis. For example, the menopausal loss of ovarian hormones is thought to contribute to AD vulnerability in women (130). Men and women have different epigenetic clocks, which affects susceptibility to diseases of aging like CVD and AD (130). Autonomic profiles also diverge, as men naturally have a higher sympathetic nervous system tone, while women have a higher parasympathetic tone (102). As a result, women, especially in younger or middle age, have stronger inflammatory responses to chronic stress, which is related to increased risk of adverse outcomes in women with CAD (102, 130). Neuropathologically, women with AD demonstrate more widespread tau tangle burden than men, suggesting distinct downstream cascades (130).

Recognizing these sex- and age-related nuances is essential for precision prevention. Tailored interventions such as aggressive midlife blood-pressure control in women or targeted vascular protection in high-risk men may yield greater reductions in both cardiovascular and cognitive morbidity than universal strategies.

## Preventative and therapeutic interventions

Understanding the role of the heart-brain axis in CVD and AD allows for greater insight into the treatment and prevention of these conditions. This is especially critical for the prevention of AD, as there are currently no treatments to effectively alter the course of the disease (131). When considering the clear pathways from CVD to AD, it becomes apparent that maintaining optimal CVH is essential to sustaining cognitive function, especially for individuals who may be genetically predisposed towards AD (10, 103).

### Lifestyle changes

Numerous behavioral changes have been found to improve health outcomes along the heart-brain axis. For example, exercise can be effective for inhibiting the inflammatory cascade and oxidative stress, particularly in women (102, 132). Physical activity enhances brain blood flow, promotes neurogenesis, and improves mood, all of which help reduce the risk of cognitive decline. Sleep deprivation is another modifiable risk factor associated with ANS disruption, increased inflammation, vascular dysfunction, and amyloid deposition, so ensuring a sufficient amount of sleep each night may confer a protective effect for cardiovascular and cognitive outcomes (13, 102). Contradictorily, a machine learning study of the UK Biobank population found sleeplessness or insomnia to be a leading protective factor against AD (133). The authors hypothesized that the most detrimental effects of sleep deprivation on cognitive decline likely occur in midlife, while longer sleep duration may occur in dementia patients later in life. Nutritional deficiencies can contribute to cognitive decline and HF, and the World Health Organization recommends that a Mediterranean diet, high in fruits, vegetables, whole gains, and omega-3 fatty acids “may help and does not hurt” risk of dementia (13, 62). Social factors are also important in promoting positive brain and heart health. Determinants such as occupational attainment, higher education, frequent social contact, and active leisure time have been found to promote maintenance of cognitive reserve despite neuropathological damage (13, 134, 135). Given the current lack of effective treatments for dementia, adopting such lifestyle interventions is advised to reduce risk.

### Pharmacological interventions

Pharmacological treatments are another area of interest. As there are not yet effective drugs to change the course of AD, a focus has emerged on modifying risk factors such as BMI, blood pressure, and lipid profiles (131). Lipid lowering and blood pressure control treatments have been shown to prevent future CVD events and delay or prevent the onset of dementia (9). Statins, commonly used for cholesterol management and cardiovascular risk reduction, are being explored for their potential neuroprotective effects. Evidence shows their use to be correlated with decreased occurrence of AD, although it is still uncertain if this is a true causal effect or the result of confounding (8). Statins may be especially beneficial for APOE4 carriers due to the allele's association with dysfunctional lipid metabolism (112). Surprisingly, despite the strong link between hypertension and AD, studies have found no conclusive evidence demonstrating that antihypertensive drugs act against dementia (8). However, some clinical trials have observed cognitive improvement in patients receiving treatment for hypotension, potentially reflecting the cognitive benefits of adequate CBF (27). Warfarin, an anticoagulant medication traditionally used to prevent forms of CVD such as stroke, myocardial infarction, and AF, has been found to reduce the incidence of all-cause dementia (136). Catheter ablation also helps control blood pressure, treat AF, and improve cognitive function, but may pose risky side effects, particularly for women (132, 137).

As inflammation is a key pathway linking heart and brain health, lifestyle modifications and pharmacological interventions are encouraged for disease prevention. Medications targeting endothelial function and inflammation are also under investigation for their ability to influence cognitive outcomes, with the aim of offering dual benefits for both heart and brain health (138). Simats et al. identified anti-inflammatory drugs that may benefit the heart-brain axis, noting that beta-blockers reduce circulating levels of inflammatory cytokines, IL-1β antibody treatment prevented cardiovascular and cerebrovascular complications, and colchicine treatment reduces stroke incidence in patients with high cardiovascular risk (139).

Control of other comorbidities can help preserve heart and brain health through inflammation reduction. Recent research highlights the value in treating depression for AD prevention. Depression is highly associated to AD incidence and often accelerates its progression through autonomic dysfunction and inflammation (140). Burke et al. found evidence suggesting mechanisms related to antidepressant usage might reduce or neutralize the hazard of eventual AD outcomes (141). Although antidepressants may play a preventative role in AD, their simultaneous effect on the cardiovascular system must be considered too. Several longitudinal studies have found evidence indicating a potential increased risk for cardiac events in long-term antidepressant users (142, 143). Evidence surrounding antidepressants emphasizes the complex long-term effects of drugs on the heart-brain axis.

Several drugs have been tested to delay the progression of AD in clinical trials. There are two major categories of AD drugs: acetylcholinesterase inhibitors (AchEIs) such as rivastigmine, galantamine, and donepezil that help facilitate the transmission of signals between nerve cells, and N-methyl-D-aspartic acid-type glutamine receptor antagonists such as memantine, which limits nerve cell damage by preventing the overexcitation of neurons (144). Both have an associated risk of severe cardiovascular side effects. AchEI usage has been associated with syncope, bradycardia, QT interval prolongation, ventricular tachycardia, and ventricular blockages, indicating that it may disrupt ANS function, while memantine is associated with a higher risk of cardiovascular events such as myocardial infarction (144). Another AD treatment involves the usage of amyloid immunotherapy, which may disrupt amyloid plaque formation and solubilize amyloid deposits, but could be accompanied by serious side effects including neuroinflammation and intracerebral hemorrhage (145).

Overall, evidence supports the treatment and prevention of CVD to limit the risk of AD and other dementias. Due to the strong links of the heart-brain axis, interventions to promote CVH or treat CVD have been shown to be protective for brain health and cognitive function. Additionally, the heart-brain connection means that future drugs for AD treatment must be carefully assessed for their impact on heart health.

## Conclusion

Future research should prioritize multidisciplinary collaboration to explore the heart-brain axis comprehensively. Combining expertise from cardiology, neurology, molecular biology, imaging, and genetics will foster a deeper understanding of this complex interplay and accelerate the translation of scientific discoveries into clinical practice (102, 138).

The intricate relationship between CVD and AD is underscored by shared pathophysiological mechanisms such as cerebral hypoperfusion, inflammation, BBB dysfunction, autonomic dysfunction, and systemic amyloidosis linking cardiovascular and cognitive health. Imaging techniques such as MRI and PET have provided deeper insights into the structural and functional connections between the heart and brain, revealing how conditions like cortical atrophy, arterial calcification, pathogenic proteins, glymphatic dysfunction, and inflammation contribute to neurodegeneration. These findings emphasize the importance of a bidirectional approach to managing CVD and AD, where addressing cardiovascular risk factors can concurrently reduce the risk of cognitive decline.

Understanding the mechanistic heart-brain pathways and their genetic and behavioral triggers is crucial for developing targeted interventions. Age-related studies stress the necessity of early cardiovascular risk management, particularly in midlife, to prevent or delay the onset of dementia. Other demographic factors such as sex must also be accounted for when assessing individual risk and tailoring prevention strategies. Current therapeutic strategies, including modulating inflammatory pathways and lifestyle modifications, show promise in mitigating the dual burden of CVD and AD.

The integration of mechanistic imaging and genetic studies provides a comprehensive understanding of the heart-brain axis, paving the way for innovative therapeutic strategies and personalized medicine approaches. By identifying specific biomarkers and mechanisms underlying heart-brain interactions, clinicians can develop tailored interventions to mitigate risks and improve outcomes for patients with cardiovascular and neurological conditions (101, 146). While current studies emphasize the existence and importance of the heart-brain axis, the complexity of these body systems necessitates further research into the pathways. Future research should systematically integrate both multi-organ imaging and genetic information in diverse populations to determine direct interventional targets. With robust support from clinical and genetic evidence, the potential for personalized medicine informed by genetic and imaging studies offers hope for more effective and precise interventions, improving patient outcomes and quality of life.
